# Complications of Botulinum Toxin, Fillers, and Threads: A Narrative Review by the Special Interest Group (SIG) Aesthetics, Indian Association of Dermatologists, Venereologists and Leprologists (IADVL) Academy

**DOI:** 10.7759/cureus.115447

**Published:** 2026-08-30

**Authors:** Rajat Kandhari, Rashmi Sharma, Ishad Aggarwal, Gulhima Arora, Nishita Ranka

**Affiliations:** 1 Dermatology, Dr. Kandhari’s Skin and Dental Clinic, New Delhi, IND; 2 Dermatology, Fortis Flt. Lt. Rajan Dhall Hospital, New Delhi, IND; 3 Dermatology, Lasers, and Aesthetics, Wizderm, Kolkata, IND; 4 Dermatology, Wizderm, Kolkata, IND; 5 Dermatology, Mehektagul Dermaclinic, New Delhi, IND; 6 Dermatology, Dr. Nishita’s Clinic for Skin, Hair and Aesthetics, Hyderabad, IND

**Keywords:** aesthetic procedures, botulinum toxin, complications, fillers, guidelines, threads

## Abstract

Aesthetic dermatology has witnessed a surge in minimally invasive procedures. While these procedures offer patient satisfaction, they also come with increased patient demands and complications, ranging from mild symptoms such as erythema, edema, and pain to more serious adverse events, including vascular compromise with fillers or infections after thread procedures. This narrative review aims to describe various complications, their prevention, and management, focusing on aesthetic injectables and threads, further serving as a guiding tool for the dermatologist to ensure safer outcomes and enhancing patient trust in cosmetic interventions.

## Introduction and background

The increasing demand for facial rejuvenation and minimally invasive aesthetic procedures has led to the widespread use of botulinum toxin, dermal fillers, and thread lifts in contemporary cosmetic dermatology. These procedures offer effective aesthetic enhancement with minimal downtime, predictable outcomes, and high patient satisfaction, making them popular alternatives to surgical interventions [1].

Botulinum toxin exerts its effect by inhibiting the presynaptic release of acetylcholine at the neuromuscular junction, resulting in temporary chemodenervation and relaxation of targeted muscles, thereby reducing dynamic facial rhytides. Dermal fillers restore facial volume, improve contours, and soften static wrinkles through tissue augmentation, while certain fillers also stimulate neocollagenesis [1]. Thread lifts employ absorbable barbed or cone-bearing sutures placed within the subcutaneous plane to provide immediate mechanical lifting of sagging tissues, with subsequent collagen remodeling contributing to longer-lasting rejuvenation [2].

Although these procedures have an excellent safety profile when performed appropriately, they are not without risk. Complications may range from mild and transient adverse events, such as bruising, edema, pain, and asymmetry, to potentially serious complications including unwanted muscle paralysis, hypersensitivity reactions, granuloma formation, vascular occlusion with tissue necrosis or vision loss, infection, thread extrusion, skin dimpling, and nerve injury [1,2].

Epidemiological data report complications with different ranges for each of the three procedures. Data suggest that botulinum neurotoxins (BoNTs) are generally considered safe for facial aesthetics; however, clinically relevant complications may occur, with reported overall rates ranging from 12% to 16% [3]. Moreover, it needs to be stressed here that, pertinently for managing blepharospasm and ophthalmological indications, the complication rate may be higher, as reported in an Indian study, up to 33.3% [4]. On the other hand, for dermal fillers, reported complication rates range from 6.5% to 32.1% [5]. Lastly, a recent meta-analysis of thread-lifting procedures reported complication rates ranging from 7% to 34% [6].

Pathophysiologically, these complications may occur due to several procedure-related and patient-related factors. They may result from incorrect site selection or excessive use of the therapeutic agent, inadvertent puncture of a blood vessel, or individual variation in the patient’s pain sensitivity threshold. Irregular or inaccurate markings may lead to improper placement of injection points, while asymmetry can arise from unequal dosing at different sites. Excessive dosing may result in unintended or prolonged muscle paralysis. Failure to assess the patient for hypersensitivity before the procedure may increase the risk of adverse reactions. Although rare, unhygienic conditions may predispose to local infection and other complications. Furthermore, the procedure requires adequate technical expertise, as improper injection technique, inaccurate anatomical localization, or inappropriate dosing can increase the likelihood of complications [7].

Current status and objectives

With the increasing use of facial rejuvenation aesthetic procedures and their expanding popularity, efficacy has been accompanied by increased publication of reported complications, which warrants wide attention, allowing for early diagnosis and management, increasing awareness, and decreasing the fear related to such complications among the patients [1].

This narrative review is designed by the Special Interest Group (SIG) Aesthetics, Indian Association of Dermatologists, Venereologists and Leprologists (IADVL) Academy, which is a dedicated professional group focused on advancing education, research, clinical practice, and patient safety in aesthetic dermatology. IADVL, established in its present form on January 28, 1973, is the national professional association representing dermatologists in India and includes more than 17,000 members. It represents specialists involved in the management of dermatological disorders, sexually transmitted infections, and leprosy. SIG Aesthetics (2026-27) brings together dermatologists with focused academic and clinical interests in aesthetic and cosmetic procedures, promoting evidence-based practice, continuing medical education, research, professional collaboration, and standardization of clinical approaches.

The narrative review aims to encompass complications associated with botulinum toxin, dermal fillers, and thread lifts, with specific objectives of synthesizing the available evidence on mechanisms, reviewing strategies for prevention and risk reduction, outlining current approaches to the recognition and management of adverse events, and identifying future directions for improving the safety and efficacy of aesthetic procedures.

## Review

Literature search strategy

A narrative literature review was conducted to synthesize the available evidence regarding complications associated with botulinum toxin, dermal fillers, and thread lifts in aesthetic dermatology, with particular emphasis on their prevention, management, underlying mechanisms, and future directions as per the Scale for the Assessment of Narrative Review Articles (SANRA) guidelines [8].

The literature search was performed using multiple electronic databases, including PubMed, DOAJ, EMBASE (Elsevier), Google Scholar, and Web of Science. The search strategy incorporated combinations of the following keywords using Boolean operators: (“adverse events” OR “complications”) AND (“botulinum toxin” OR “dermal fillers” OR “thread lifts”). Relevant articles published in English between 2011 and 2026 were considered. Observational studies, cohort studies, systematic and narrative reviews, consensus statements, and clinical guidelines were considered based on their relevance to the objectives of the review. Articles unrelated to aesthetic applications, duplicate publications, non-English articles, articles reported on animals, and publications lacking sufficient information relevant to the review objectives were excluded.

As this was designed as a narrative review, the selection of literature was based on relevance, clinical applicability, and the available level of evidence rather than a formal systematic review screening protocol. Therefore, a PRISMA-based study selection process, quantitative synthesis/meta-analysis, and formal risk-of-bias assessment were not undertaken. However, the search period, databases, search terms, language restriction, and eligibility criteria have been specified to improve transparency and reproducibility.

Botulinum toxin

Botulinum toxin injections remain a cornerstone of management for muscle paralysis to reduce the probability of aging lines. However, its action may spread to adjacent muscles, causing complications due to the compact spatial arrangement of muscles, anatomical variations, injection technique, or product diffusion [1,9-11].

BoNT administration may be associated with a range of adverse events, most of which are localized to the injection site or treated muscles, although systemic adverse events may occasionally occur [12]. Among systemic events, patients may experience non-specific symptoms such as flu-like symptoms, headache, generalized weakness, fatigue, and malaise, which are generally transient and self-limiting. Hypersensitivity or allergic reactions are uncommon but may present with localized or generalized manifestations and require prompt recognition and appropriate management. A rare but potentially serious complication is systemic spread of botulinum toxin beyond the intended injection site, which may result in distant muscle weakness and neurological manifestations. Depending on the extent of toxin spread, patients may develop dysphagia, dysarthria, diplopia, generalized muscle weakness, or respiratory compromise. The risk may be influenced by factors such as the dose administered, injection technique, anatomical site, and patient susceptibility. Although these complications are uncommon, clinicians should counsel patients regarding relevant warning symptoms and ensure prompt evaluation when progressive generalized weakness, difficulty swallowing, or respiratory symptoms develop [12]. The localized complications associated with BoNT administration for facial aesthetics have been divided into those related to the upper face and lower face [1,9-11].

Neurotoxin-Related Adverse Effects of the Upper Face and Their Management

BoNT administration to the upper face may be associated with a range of localized adverse events. These complications are generally related to the pharmacological effect of BoNT, inadvertent diffusion to adjacent muscles, injection technique, dose, anatomical variation, or individual patient characteristics. The commonly recognized complications include headache, Mephisto effect or “Spock” brow, eyelid ptosis, brow ptosis, and cheek ptosis [1,2].

Headache: Headache following BoNT administration is generally transient and self-limiting. It may be related to injection-related discomfort, transient changes in muscle activity, or individual susceptibility. Symptoms are usually mild and resolve within a few days, although occasionally more severe or prolonged headache may occur. Management is primarily conservative and includes reassurance, cold compresses, gentle massage, and systemic analgesics such as ibuprofen when required. An atraumatic injection technique and minimizing procedural discomfort may help reduce its occurrence [1].

Mephisto effect or spocking: The “Spock” brow, characterized by an unnaturally elevated lateral eyebrow (Figures 1A-1D), is a recognized complication of BoNT treatment and results from an imbalance between the muscles responsible for eyebrow elevation and depression. It commonly occurs when the medial portions of the frontalis are weakened disproportionately, allowing relatively greater activity of the untreated lateral frontalis fibers. Excessive treatment of the brow depressors, including the corrugator supercilii, procerus, and orbicularis oculi, without adequate consideration of frontalis activity may further contribute to this appearance [9].

**Figure 1:**
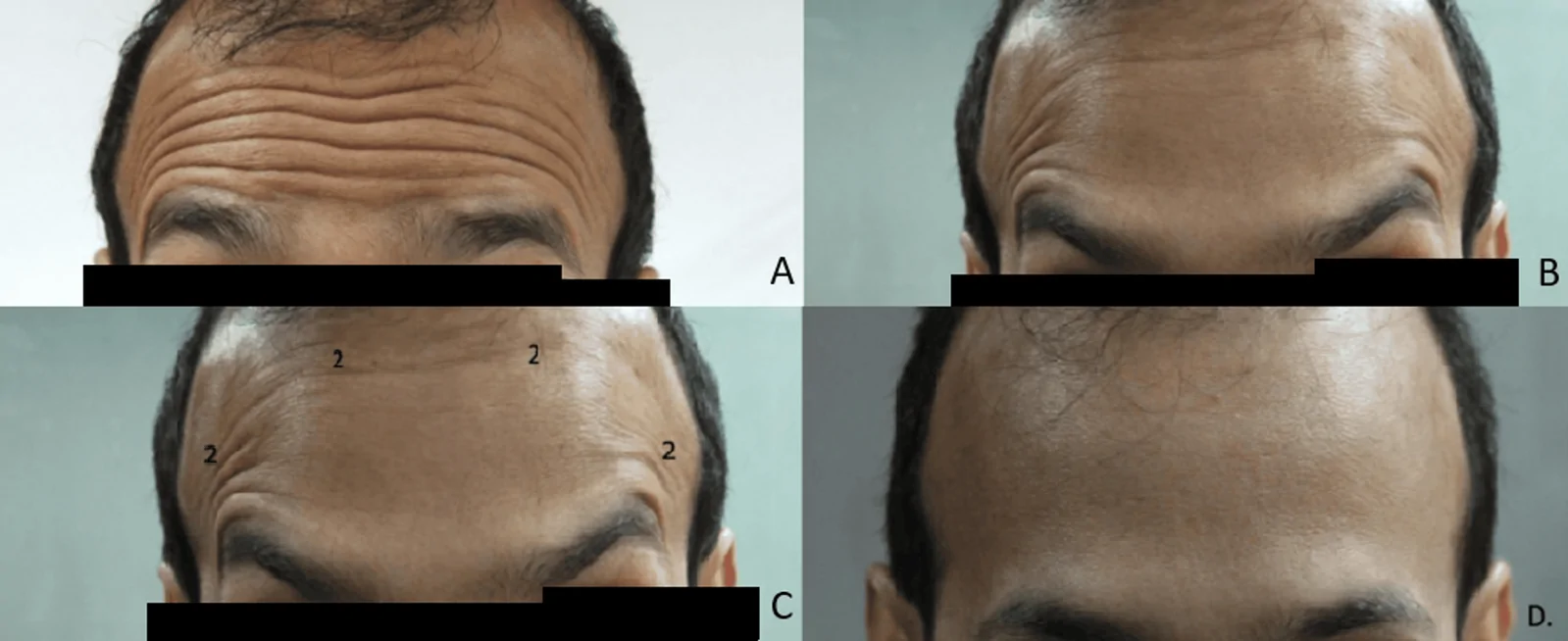
Figure showing the Mephisto effect. (A) Upper forehead expression lines on animation before treatment with botulinum toxin. (B) Visible spocking post-treatment with botulinum toxin. (C) Addressing the spocking with 2 units each of botulinum toxin at the illustrated sites. (D) Patient after Botox treatment. The patient provided written informed consent for the use and publication of this photograph in scientific and educational publications.

Prevention of the "Spock" brow begins with identifying patients predisposed to lateral frontalis overactivity ("Spockers") during the baseline assessment (Figure 2).

**Figure 2:**
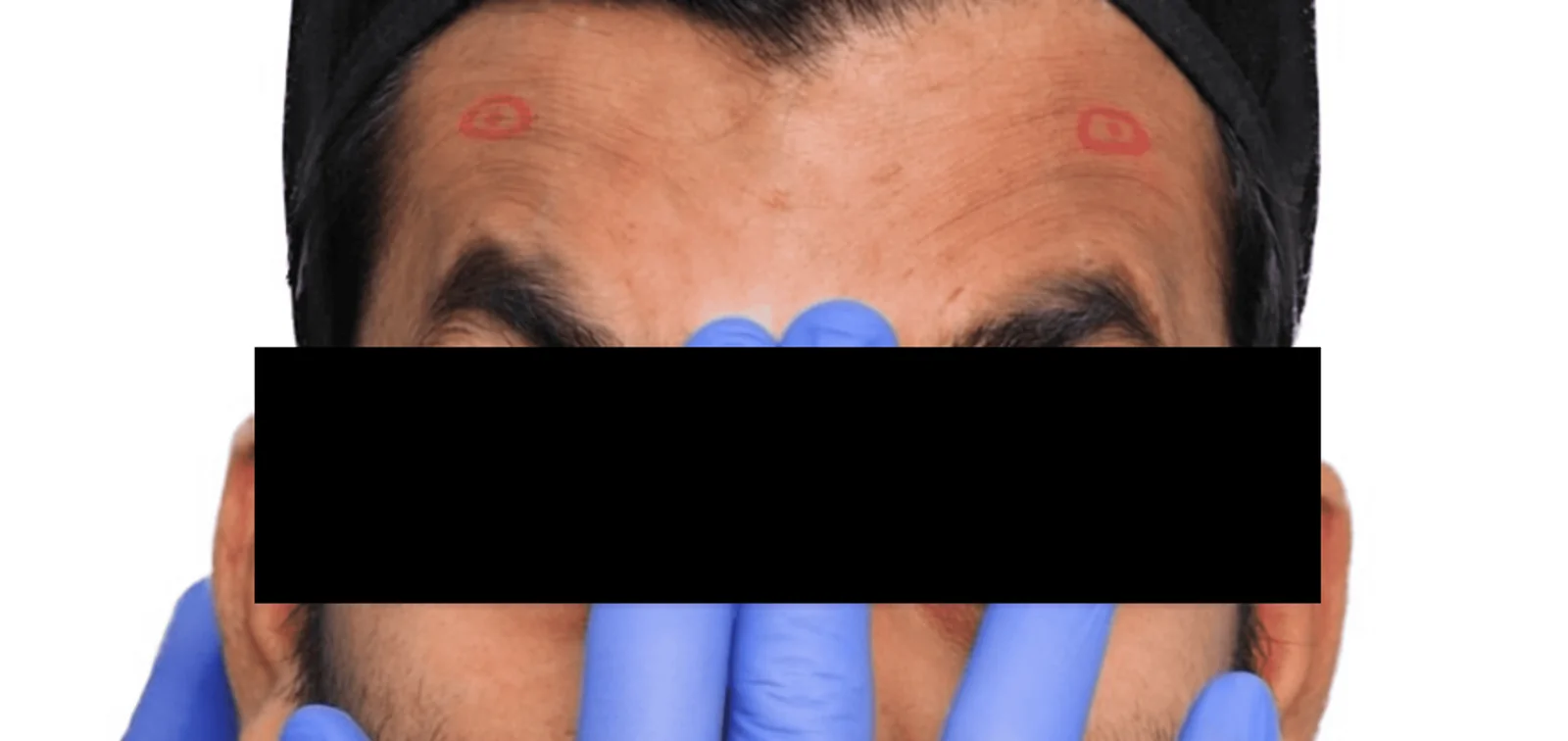
Patient photo demonstrating the anti-Spock point (red circles) by blocking the depressors and asking the patient to raise their brows. Written informed consent for publication of the clinical image was obtained from the patient.

Careful evaluation of frontalis muscle dynamics enables appropriate balancing of botulinum toxin units across the frontalis muscle, thereby minimizing excessive lateral brow elevation and maintaining a natural eyebrow contour. In patients who develop a persistent Mephisto effect, a small targeted corrective injection into the lateral frontalis at the point of maximal brow elevation may be considered, followed by reassessment after approximately 7-10 days [9].

Eyelid ptosis: It occurs when BoNT diffuses into the levator palpebrae superioris muscle (LPS) via the supraorbital notch [13]. The LPS is the primary retractor of the upper eyelid. Close to 5% of individuals treated for the upper face experience eyelid ptosis, and it develops 2-10 days post-injection. It can persist for 2-4 weeks and is self-limiting [10,11].

Ptosis is detected by assessing the degree of upper eyelid movement during upward gaze, a palpebral fissure distance of less than 7 mm, and a marginal reflex distance of less than 4 mm. The clinician should document with photographs and differentiate primary lid ptosis and secondary blepharoptosis in individuals with pre-existing lid laxity (Figures 3A-3E). Alpha-adrenergic agonists help in retraction of the upper lid [13,14]. Prevention involves appropriate injection depth and dose, particularly around the corrugator and periorbital region, with injections directed away from the orbit and avoidance of excessive diffusion [13].

**Figure 3:**
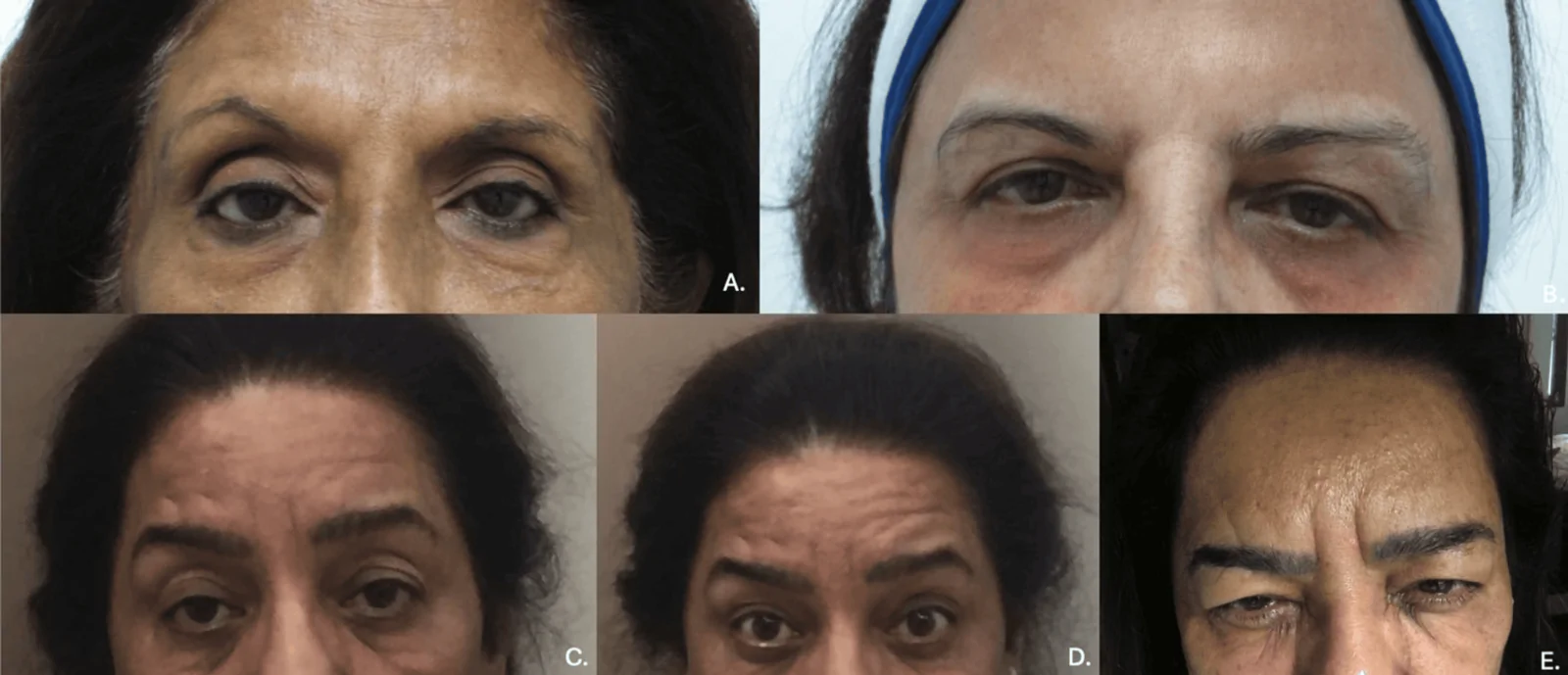
Practice caution in patients with (A) eyebrow asymmetry with lid laxity and blepharochalasis, (B) pre-existing brow ptosis with blepharochalasis, (C) pre-existing lid laxity and compensatory action of a hyperkinetic frontalis, (D) pretreatment brow elevation with botulinum toxin, and (E) pseudoblepharoptosis after treatment, due to compromised movement of the frontalis muscle and worsening of the lid laxity, simulating a lid ptosis. Written informed consent for publication was obtained from the patients.

Brow ptosis: Brow ptosis generally results from excessive weakening of the frontalis, leading to loss of the muscle's brow-elevating support. It may occur following aggressive treatment of the frontalis, particularly when injections are placed too close to the supraorbital ridge or when lateral frontalis fibers are excessively weakened. Diffusion of BoNT from treatment of the brow depressors may also affect the low-lying medial fibers of the frontalis and contribute to medial brow descent [15-18].

Prevention requires assessment of baseline brow position, frontalis activity, and the patient's dependence on frontalis contraction for maintaining eyebrow elevation. Appropriate injection placement and conservative dosing are important, and the Convergence Line concept may assist in determining safe injection boundaries (Figure 4). When brow ptosis occurs, treatment is generally conservative, with reassurance and observation because spontaneous recovery is expected as neuromuscular function returns [18].

**Figure 4:**
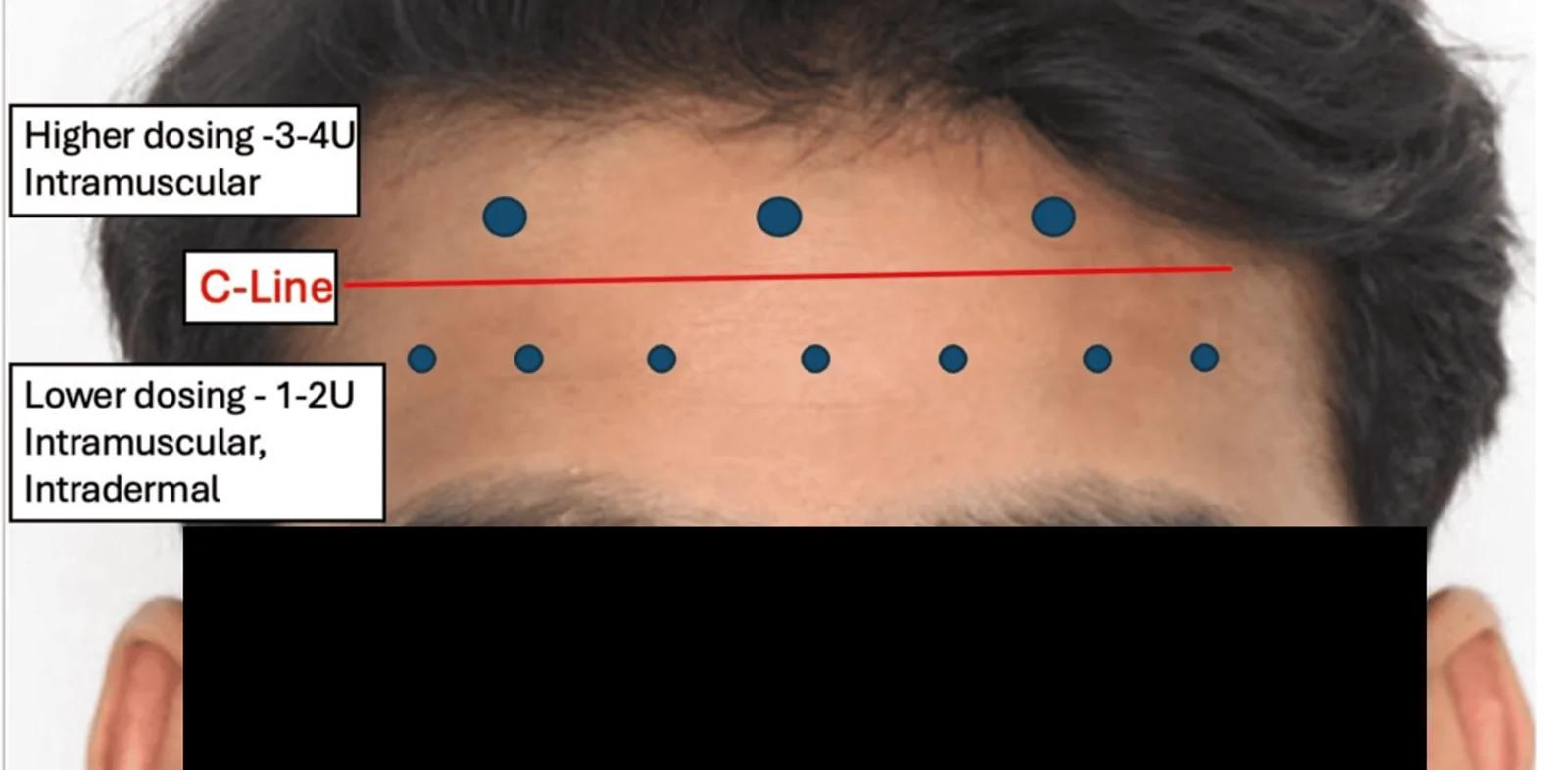
Demonstration of the C-line technique. Higher doses are administered above the C-line using an intramuscular injection plane, whereas lower doses are placed below the C-line using an intradermal injection technique. Written informed consent for publication was obtained from the patient.

Cheek ptosis: Cheek ptosis may occur when BoNT inadvertently affects muscles involved in cheek elevation, particularly the zygomaticus major and minor, during treatment of lateral canthal lines. To minimize this risk, injections should be placed superficially and at an appropriate distance from the orbital rim, with careful consideration of the underlying facial anatomy [10]. Mild cases are generally managed conservatively with observation and reassurance until neuromuscular function recovers. In selected patients with persistent alteration of cheek contour, dermal fillers may be considered to restore volume and support facial contour [19].

Others: Other less frequent adverse effects may include diplopia or strabismus, lagophthalmos, xerophthalmia, and eyelid edema. Diplopia or strabismus may result from inadvertent spread of BoNT to extraocular muscles and is generally managed conservatively, although ophthalmological assessment may be warranted in persistent or significant cases. Excessive inhibition of the orbicularis oculi may contribute to lagophthalmos and ocular surface symptoms; these may be managed with lubricating eye drops or ointments and, when necessary, additional measures to protect the ocular surface. Eyelid edema is generally self-limiting and may be managed conservatively with head elevation and measures that facilitate venous and lymphatic drainage. Appropriate patient selection, conservative dosing, accurate anatomical localization, and meticulous injection technique are important preventive measures for these complications. The risk of these adverse events can be minimized through careful pre-procedure assessment, individualized dosing, precise anatomical localization, appropriate injection technique, and adequate knowledge of facial anatomy [20].

Neurotoxin-Related Adverse Effects of the Lower Face and Their Management

Botulinum toxin treatment of the lower face requires careful consideration of the close anatomical relationships between the muscles responsible for facial expression, mastication, and oral competence. Adverse effects may occur because of unintended diffusion to adjacent muscles, inaccurate injection placement, excessive dosing, or inadequate assessment of individual facial anatomy. Important complications include lip ptosis, asymmetric smile, masseteric bulging or “popping,” oral sphincter incompetence, and jowling or lower-face sagging [1,21,22].

Lip ptosis: Lip ptosis may occur when BoNT unintentionally diffuses to muscles responsible for upper-lip elevation, including the levator labii superioris alaeque nasi (LLSAN), levator labii superioris (LLS), levator anguli oris (LAO), or zygomaticus muscles. Clinically, it may manifest as asymmetry or reduced upper-lip movement, particularly during smiling. Diffusion to the LLS or zygomaticus muscles during treatment of a gummy smile may result in upper-lip ptosis or flattening of the nasolabial fold, whereas involvement of the LLSAN may occur during treatment of the nasalis muscle [21,23].

Prevention depends on accurate identification of the target muscle, careful attention to anatomical landmarks, appropriate injection depth, and conservative dosing. When lip ptosis occurs, management is generally conservative, with observation and reassurance until neuromuscular function returns [21,23].

Asymmetric smile: Asymmetric smile is an uncommon but aesthetically significant complication of BoNT treatment and results from unintended weakening of muscles involved in oral commissure movement. During treatment of the depressor anguli oris (DAO), inadvertent diffusion to the depressor labii inferioris (DLI) may cause asymmetry of the lower lip. Similarly, during treatment of masseteric hypertrophy, risorius diffusion via anteriorly placed/superficial masseter injection may reduce lateral excursion of the oral commissure and produce a characteristic “crooked” smile [22].

The risk may be increased by superficial or excessively anterior injections and by inadequate assessment of masseter anatomy. Prevention therefore requires careful patient selection, accurate identification of the target muscle, appropriate injection depth and placement, and conservative dosing. Most cases are self-limiting and can be managed with observation and reassurance, with corrective treatment considered only in persistent or clinically significant cases [22].

Popping of the deep head of the masseter: Masseteric “popping” or bulging may occur when BoNT produces inadequate or asynchronous weakening of the different heads of the masseter. When the superficial head is preferentially weakened while the deeper portion remains relatively active, the deeper component may become visibly prominent during clenching, producing a characteristic bulge or “chipmunk” appearance. This can be recognized clinically by palpating the masseter during maximal dental clenching [1,22].

Prevention requires appropriate assessment of masseter anatomy and treatment of the muscle at suitable depth to achieve balanced neuromuscular weakening. A sufficiently long needle may be required to reach the deeper component when clinically indicated [1,22]. If persistent, carefully targeted additional treatment may be considered after reassessment.

Popping of the superficial head of the masseter: The converse phenomenon may occur when the deep head of the masseter is adequately weakened but the superficial head remains relatively active. This may result from inadequate toxin diffusion to the superficial component, potentially related to the internal architecture of the masseter, including the deep inferior tendon. Clinically, the superficial head may remain palpable or become visibly prominent during clenching [1,22].

Prevention involves appropriate assessment of both superficial and deep components of the masseter and consideration of injection at appropriate depths to achieve more uniform treatment. When clinically significant residual prominence persists, cautious supplementary treatment of the superficial component may be considered following reassessment. Most cases are self-limiting as the effects of BoNT evolve [1,22].

Oral sphincter incompetence: Inadvertent diffusion of BoNT to the orbicularis oris may result in temporary oral sphincter incompetence, with functional manifestations such as difficulty with lip closure, drinking, speech, or oral competence. This complication may occur following excessive dosing or high-volume injections in the perioral region, including during treatment of the DAO, mentalis, or LLSAN [1,3].

Because oral sphincter incompetence may be functionally significant, prevention is particularly important [3]. Conservative dosing, precise injection placement, appropriate injection depth, and avoidance of excessive manipulation or massage of the treated area can help minimize unintended diffusion. Once established, management is primarily supportive, with reassurance and observation until neuromuscular function recovers [1,3].

Jowling and lower-face sagging: Jowling or sagging of the overlying skin may become more apparent following excessive weakening of the masseter, particularly in patients with pre-existing skin laxity or reduced soft-tissue support. Reduction in masseteric volume may alter lower-face contour and accentuate pre-existing laxity [24].

Prevention requires appropriate patient selection and conservative dosing, particularly in individuals with significant pre-existing skin laxity or jowling. Patients should be counseled regarding the possibility of altered lower-face contour before treatment. When this occurs, management is generally conservative, with observation as the neuromuscular effect diminishes and the facial contour stabilizes [24].

Fillers

The rapid growth in the use of hyaluronic acid fillers (HAFs) for soft-tissue augmentation over the last few years has also witnessed an increase in the number of complications being seen due to filler use [25]. Complications of dermal fillers are caused mainly by injection technique, filler characteristics, or host immune responses. Immediate erythema and edema are generally due to local tissue trauma, while superficial filler placement may additionally contribute to persistent swelling. Ecchymosis is caused by perforation or rupture of dermal vessels during injection. Malar edema, particularly after infraorbital augmentation, is caused when filler exerts pressure on lymphatic vessels in the setting of compromised lymphatic drainage [25,26].

Hypersensitivity reactions may be type I, mediated by immunoglobulin E and mast cells, or delayed type IV reactions, mediated mainly by T lymphocytes. Delayed reactions may manifest as erythema, edema, pruritus, induration, or sterile abscess-like lesions. Superficial placement of hyaluronic acid (HA) can cause the Tyndall effect by scattering of blue light [25].

Nodules may be caused by excessive filler, superficial placement, inappropriate product selection, infection, biofilm formation, hypersensitivity, or foreign-body reactions. Biofilms may develop when bacteria introduced from the skin surface during injection colonize the filler and subsequently trigger infection or chronic inflammation. Foreign-body granulomas may occur when filler resists enzymatic degradation or phagocytosis, leading to macrophage accumulation, cytokine release, and formation of multinucleated giant cells [25].

Filler migration may develop due to high injection volumes, excessive pressure, massage, gravity, lymphatic spread, or intravascular injection. The most serious complication, vascular occlusion, is caused by inadvertent intra-arterial injection or compression of adjacent vessels, potentially leading to tissue ischemia and necrosis. In the retinal circulation, embolization can lead to obstruction of retinal arteries and develop visual loss [25].

Skin Discoloration

Bruising and ecchymosis: Bruising and ecchymosis following HAF injections are common, particularly in highly vascular areas such as the periorbital region and lower face and in individuals with thin skin [27]. Patients should be counseled regarding the possibility of bruising, particularly when treatment is planned before important social or professional events, and elective procedures may preferably be avoided within two weeks of such events. Vessel injury during injection is the principal mechanism. The risk can be minimized by using an atraumatic technique, applying firm pressure following injection, minimizing the number of punctures, using fine 27-30-gauge needles or an appropriate cannula, and injecting slowly. Importantly, prescribed antiplatelet or anticoagulant medications should not be routinely discontinued solely to facilitate an elective aesthetic procedure, as the potential systemic risks of interruption may outweigh the cosmetic benefit [27,28].

Most bruises are self-limiting. Cold compresses may be applied during the first 24 hours, followed by warm compresses after 24 hours to facilitate resolution. Topical vitamin K cream for 7-10 days may be considered. Arnica, either topical 30C or oral 200 mg three times daily, and bromelain 400-500 mg, initiated approximately two days before the procedure and continued for seven days, have also been described for reducing bruising, although the strength of evidence supporting these measures varies [27,28].

Neovascularization: Neovascularization or persistent telangiectatic change at the filler-treated site may occur because of neoangiogenesis or pressure-related effects on dermal vessels [27,28]. Appropriate filler selection, accurate injection depth, and avoidance of excessive filler deposition may reduce this risk. Persistent vascular changes may be treated using vascular-targeted modalities such as potassium titanyl phosphate (KTP) laser or intense pulsed light (IPL), depending on the clinical presentation [27,28].

Post-inflammatory hyperpigmentation: Post-inflammatory hyperpigmentation (PIH) following filler injections has been reported more frequently or may be more clinically apparent in individuals with higher Fitzpatrick phototypes [28]. It may result from post-traumatic inflammation following multiple needle passes or tissue injury. An atraumatic injection technique and avoidance of repeated passes may reduce the risk. Most cases gradually resolve; however, persistent pigmentation may be treated with topical depigmenting agents, while resistant cases may be considered for Q-switched Nd:YAG laser treatment [29].

Tyndall effect: Tyndallization occurs when HAF is placed too superficially within the skin. The deposited HA creates an optical environment in which longer wavelengths of light are preferentially absorbed while shorter wavelengths are scattered and reflected, producing a characteristic bluish discoloration [30]. The effect is particularly evident when excessive filler is deposited superficially, and the more superficial the placement, the longer the discoloration may persist [31]. The periorbital region is particularly susceptible because of its thin skin [32].

Prevention depends on appropriate product selection and placement at the correct anatomical depth, with avoidance of excessive superficial filler deposition. When a clinically significant Tyndall effect develops, hyaluronidase (HYAL) may be used to dissolve HAF. In selected superficial collections, an 18-gauge needle may be used to puncture and express the excess filler [31,32].

Edema

Edema after HAFs can be immediate in onset, or it can be a delayed occurrence [25].

Immediate post-filler edema: Edema following HAF injection may be immediate or delayed [33]. Some degree of immediate swelling is expected after filler placement, with severity influenced by the injection site, amount and physicochemical properties of the filler, tissue characteristics, and injection technique. The lips and infraorbital region are particularly prone to swelling, while highly hygroscopic fillers may produce greater edema. Excessive injection force and overcorrection may further increase swelling. Gentle injection technique, appropriate product selection, and avoidance of overfilling may reduce the severity. Most uncomplicated post-procedural edema resolves within approximately one week and can be managed with reassurance, cold compresses, and, where clinically appropriate, oral antihistamines or anti-inflammatory agents [33].

Angioedema or edema due to histamine release: Acute angioedema or histamine-mediated edema may develop within approximately one hour of injection and may be associated with pruritus and urticarial wheals [34]. Such reactions may represent an immediate hypersensitivity response. Patients should be assessed for systemic involvement, particularly airway compromise or respiratory symptoms. Management may include oral antihistamines with or without a short course of systemic corticosteroids, depending on severity [34].

Delayed hypersensitivity reaction: Delayed hypersensitivity reactions may develop from the first day to several weeks following treatment and may present with swelling, erythema, induration, or tenderness. These reactions may occur in response to HA itself or other filler components, including the cross-linking agent 1,4-butanediol diglycidyl ether (BDDE). Delayed inflammatory reactions have also been reported in association with influenza-like illnesses, upper respiratory tract infections (URTIs), COVID-19 infection, and COVID-19 vaccination in previously treated patients. Persistent reactions may be poorly responsive to antihistamines or systemic corticosteroids and, in patients treated with HAFs, may require HYAL administration [35].

Malar edema: Malar edema is particularly associated with infraorbital filler placement and is thought to result from blockage of lymphatics and impermeability of the malar septum [36]. It may develop within several days after injection and can persist for prolonged periods. Prevention includes careful patient selection, avoidance of filler placement in patients with prominent malar bags or pre-existing edema, and cautious product selection. Initial management is generally conservative; persistent edema lasting more than 3-4 weeks may be considered for HYAL treatment when HAF is implicated [36]. The protocol for the management of edema post-HAF is shown in Figure 5.

**Figure 5:**
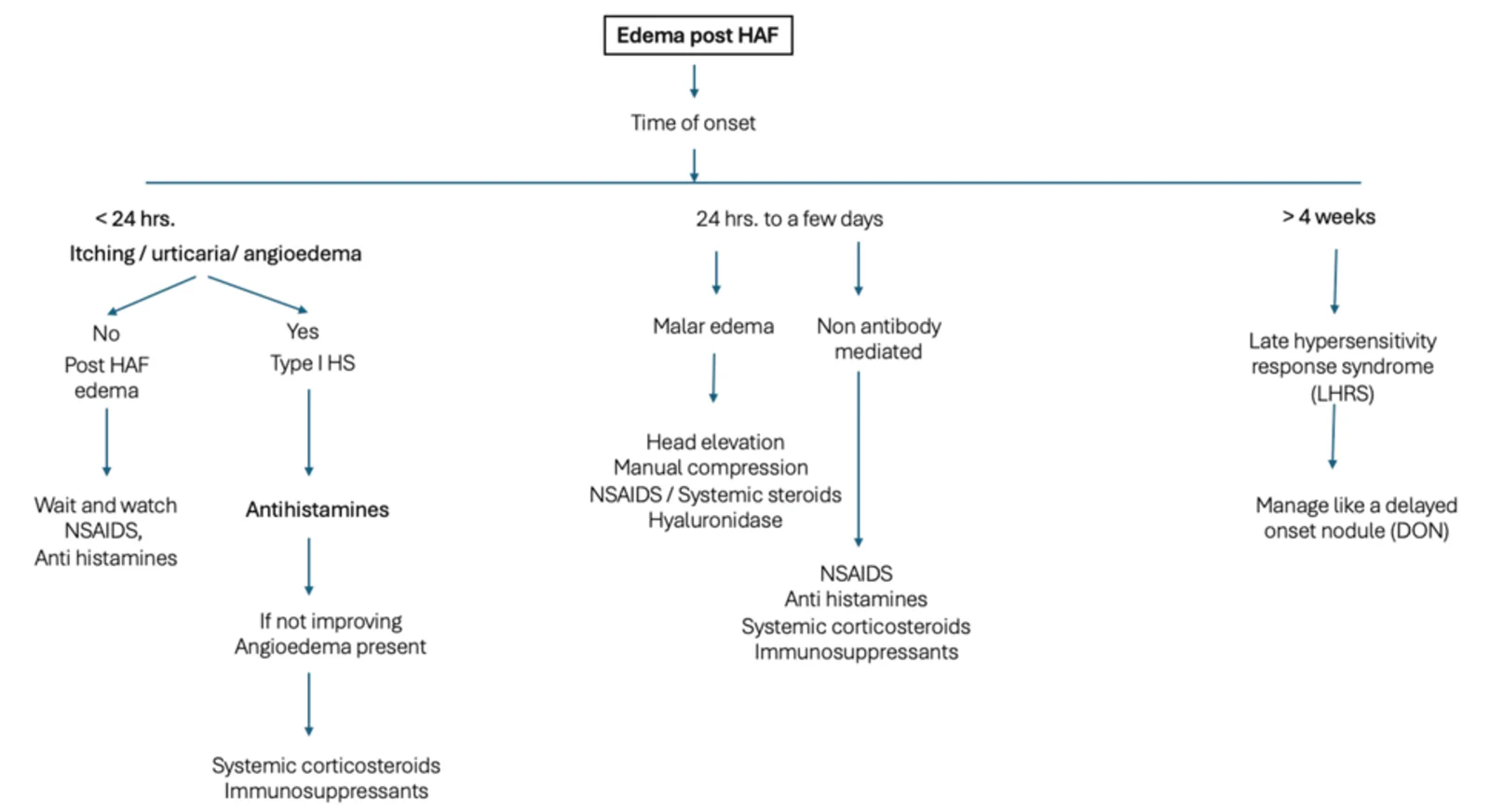
Stepwise management of edema post-HAF. NSAIDs: non-steroidal anti-inflammatory drugs; HAF: hyaluronic acid filler; HS: hypersensitivity

Lumps and Nodules

Lumps and nodules are relatively common following HAF injections and can be classified according to their inflammatory status or time of onset [37,38].

Non-inflammatory lumps: They are typically caused by incorrect injection technique or inappropriate product placement, such as using the wrong filler or placing it too superficially. Other contributing factors include hyperkinetic muscles, which may displace the filler, and excessive filler volume injected into a small anatomical area. Prevention involves appropriate product selection, accurate placement within the intended tissue plane, and use of conservative volumes and small aliquots. Management depends on the underlying cause and may include gentle molding of the filler, administration of HYAL to dissolve HAFs, or evacuation of the filler through a small stab incision using an 18-gauge needle [37,38].

Inflammatory nodules: Inflammatory nodules are usually delayed-onset nodules (DONs) presenting after four weeks to even more than a year after treatment. Delayed inflammation or late hypersensitivity response syndrome (LHRS) is manifested by a chronic granulomatous inflammation, which presents as tender, painful nodules at the site of injection [39], previously described as “angry red bumps,” a descriptive clinical phenotype rather than a distinct diagnostic entity [40]. Factors such as genetic predisposition, filler composition, HA chain length, underlying infections (particularly URTI, sinusitis, etc.), and biofilms may be responsible [39-43]. While inflammatory nodules can be diagnosed clinically, they warrant a skin biopsy and a pus culture, gram stain, and stain for acid-fast *Bacillus* (AFB) and are usually managed with a course of steroids and antibiotics [43]. A patient who developed inflammatory nodules is shown in Figure 6, which showed resolution of the inflammatory nodules with the course of steroids and antibiotics.

**Figure 6:**
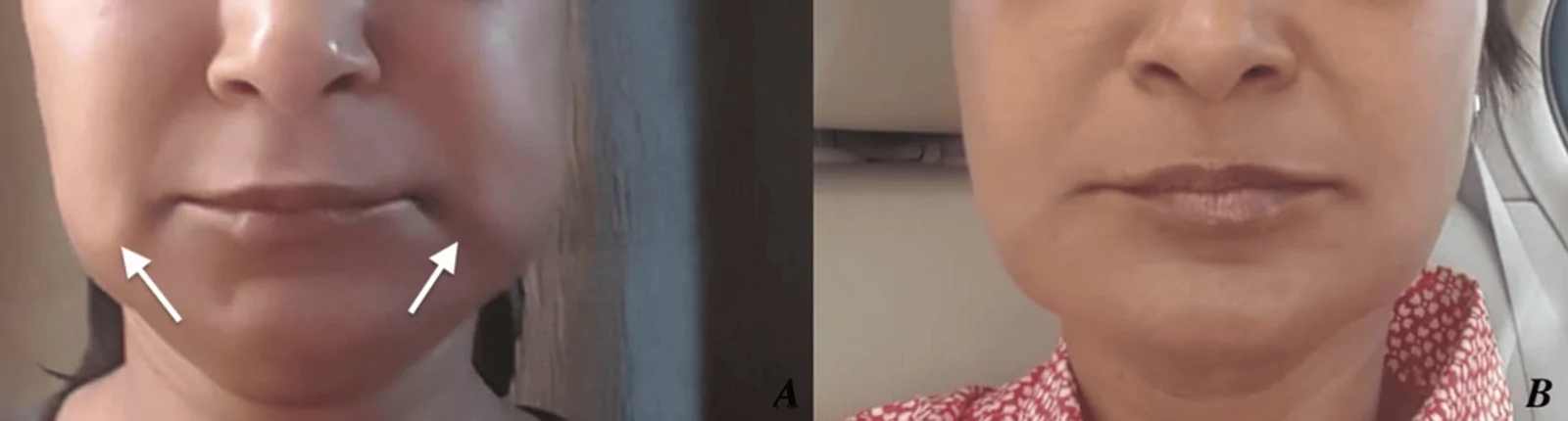
Inflammatory nodule. (A) White arrow shows the nodules at the site of injection and diffuse swelling after injecting HAFs. (B) Post-treatment with a course of steroids and broad-spectrum antibiotics. HAFs: hyaluronic acid fillers Written informed consent for publication was obtained from the patient.

Management should be individualized according to the underlying mechanism and may include oral antibiotics when infection or biofilm-related inflammation is suspected, systemic corticosteroids for significant inflammatory reactions, HYAL for HAFs, and, in refractory cases, intralesional corticosteroids with or without 5-fluorouracil (5-FU), non-steroidal anti-inflammatory drugs (NSAIDs), or surgical excision [39-43]. A stepwise approach for management of delayed inflammatory nodules has been shown as a flowchart in Figure 7.

**Figure 7:**
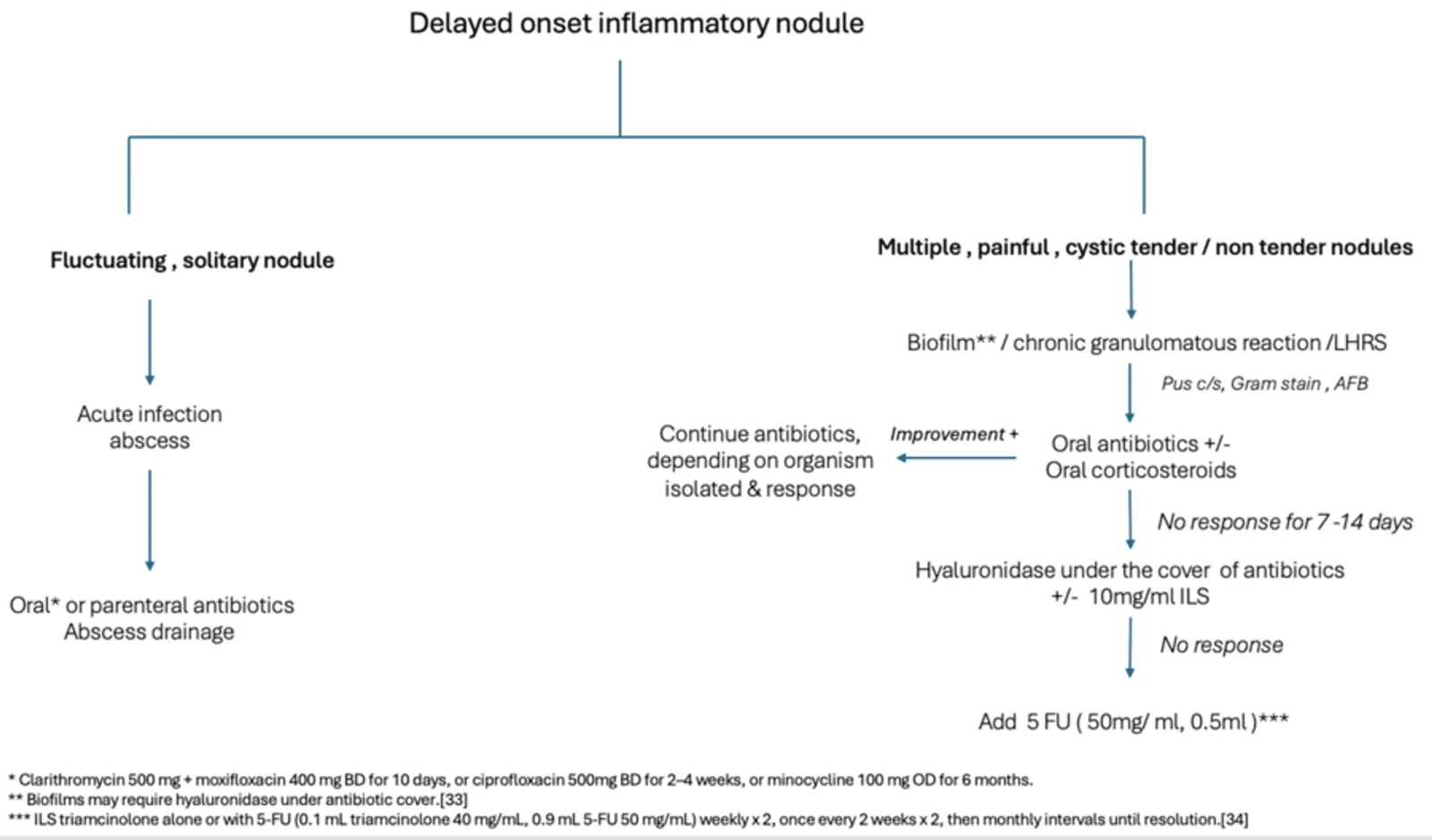
Algorithm for the management of delayed-onset inflammatory nodules. LHRS: late hypersensitivity reaction syndrome; c/s: culture and sensitivity; AFB: acid-fast bacilli; ILS: intralesional steroid; 5-FU: 5-fluorouracil; BD: twice daily; OD: once daily

Infections

Infections following HAF injections may be bacterial or viral. Bacterial infections, commonly involving *Staphylococcus aureus* and *Streptococcus pyogenes*, may present as cellulitis, erysipelas, or fluctuant abscesses [44]. Prevention depends on strict aseptic technique, appropriate skin preparation, avoidance of injection through actively inflamed or infected skin, and appropriate post-procedural care.

Mild bacterial infections may be treated with oral antibiotics such as amoxicillin/clavulanate 625 mg three times daily or clindamycin 600 mg three times daily for 7-10 days, depending on the clinical presentation and local antimicrobial guidance. More severe infections may require parenteral antibiotics, while abscesses require appropriate drainage [44].

Herpes simplex virus (HSV) reactivation may also occur following filler injections. In patients with a history of recurrent herpes simplex infection, antiviral prophylaxis may be considered; the regimen described in the literature includes valacyclovir 500 mg twice daily for three days. Active HSV infection should be treated according to standard antiviral treatment protocols [44].

Vascular Compromise

Vascular compromise is one of the most serious complications associated with HAF injections and may result from either direct intravascular injection or extravascular compression of blood vessels [45]. Depending on the vascular territory involved, complications may range from localized skin ischemia and tissue necrosis to retinal artery occlusion, visual loss, diplopia, ophthalmoplegia, and, rarely, neurological complications. Early recognition and immediate intervention are critical to minimize irreversible tissue or visual injury [45].

Peripheral ischemia with impending necrosis: The earliest manifestations of peripheral vascular compromise include disproportionate or severe pain and changes in skin color. Arterial occlusion is typically characterized by severe, immediate pain, whereas venous occlusion may present with less severe and more delayed pain associated with bluish discoloration resulting from venous congestion [45,46]. The affected skin may show blanching and a livedoid or reticular pattern. Over the subsequent 1-2 days, blistering and ischemic pustules may develop, indicating progressive tissue ischemia and impending necrosis. Ulceration and irreversible tissue necrosis may subsequently occur [45,46].

Importantly, transient pallor or blanching immediately after injection should be distinguished from true vascular compromise, particularly when local anesthetic containing a vasoconstrictor has been used. Local anesthetic-related vasoconstriction may produce temporary, relatively uniform pallor that generally resolves as the anesthetic effect wears off and is not accompanied by progressive pain, livedoid discoloration, delayed capillary refill, blistering, or tissue breakdown. In contrast, filler-related arterial compromise is more likely to present with disproportionate or escalating pain, persistent or mottled blanching, delayed capillary refill, livedoid or reticular changes, and subsequent dusky or violaceous discoloration. Persistence or progression of these findings should raise immediate suspicion of vascular occlusion and prompt urgent intervention [45,46].

If vascular compromise is suspected, the injection should be stopped immediately and measures to restore blood flow initiated without delay [45]. Prevention includes detailed knowledge of facial vascular anatomy, appropriate patient selection, conservative injection volumes, slow injection with low pressure, use of small aliquots, and appropriate selection of needles or cannulas in high-risk areas. Aspiration may be considered as an additional precaution, although a negative aspiration does not reliably exclude intravascular placement [45].

For HAF-related vascular occlusion, high-dose pulsed HYAL protocols have been described. One approach involves approximately 500 units per affected area, with up to 1,500 units when three areas are involved, with reassessment and repeat administration every 60-90 minutes until capillary refill improves to less than approximately 4 s, with treatment completed within 72 hours [45,46]. Warm compresses may be used to promote vasodilation and perfusion. Antiplatelet therapy, such as an initial aspirin dose of 500 mg followed by 75-100 mg daily for approximately one week, has also been described, although treatment should be individualized according to the patient's clinical status and contraindications [45,46].

Retinal and ocular vascular complications: retinal artery occlusion, vision loss, diplopia, and ophthalmoplegia: Retinal and ocular vascular complications represent a distinct and potentially devastating manifestation of filler-related vascular compromise and warrant immediate emergency evaluation. Retinal artery occlusion can happen due to retrograde flow of filler in the ophthalmic artery, characterized by intense eye pain, blurring of vision, ophthalmoparesis, and/or visual loss. Filler injections anywhere in the territory of the facial artery could retrogradely be injected into the ophthalmic artery to occlude it [47]. A filler embolus in the central retinal artery circulation can cause irreversible and permanent blindness within 60-90 minutes, and retinal necrosis ensues subsequently. Injection pressures above the systolic arterial pressure were needed to transfer filler into the ophthalmic artery (166.7 mm Hg). As little as 0.085 mL (range, 0.04-0.12 mL) can result in an embolus and occlusion, and the filler can disperse to multiple vessels and travel retrogradely to the orbit or internal carotid artery and cerebral circulation, causing CNS complications, and/or distally to the smaller branches supplying the skin [48].

While vision loss itself can be painless, patients can experience severe pain at the injection site, ecchymosis in the eyes, and symptoms of intracranial embolization such as vertigo and headaches [49]. High‐risk areas include the glabella, nasal region, nasolabial fold, forehead, and temple area [50]. A classification and prognosis of periocular complications related to blindness after filler injections has been suggested, dividing scenarios into four categories involving partial and complete loss of vision, ptosis, and/or ophthalmoplegia [51]. Occlusion of the ophthalmic artery leads to painful vision loss; central retinal artery occlusion leads to painless vision loss, while posterior ciliary artery occlusion may lead to partial vision loss and/or field loss. Most cases of blindness are unilateral, and few are amenable to recovery. Early recognition and treatment are essential to fully or partially regain vision [52]. It is important to connect with an ophthalmologist to consider administering retrobulbar HYAL [53,54]. Although retrobulbar HYAL remains a key treatment option, its effectiveness varies and necessitates optimization [55]. A stepwise approach to the management of vascular compromise, including the reconstitution and administration of HYAL, is illustrated in Figures 8, 9 [48,56].

**Figure 8:**
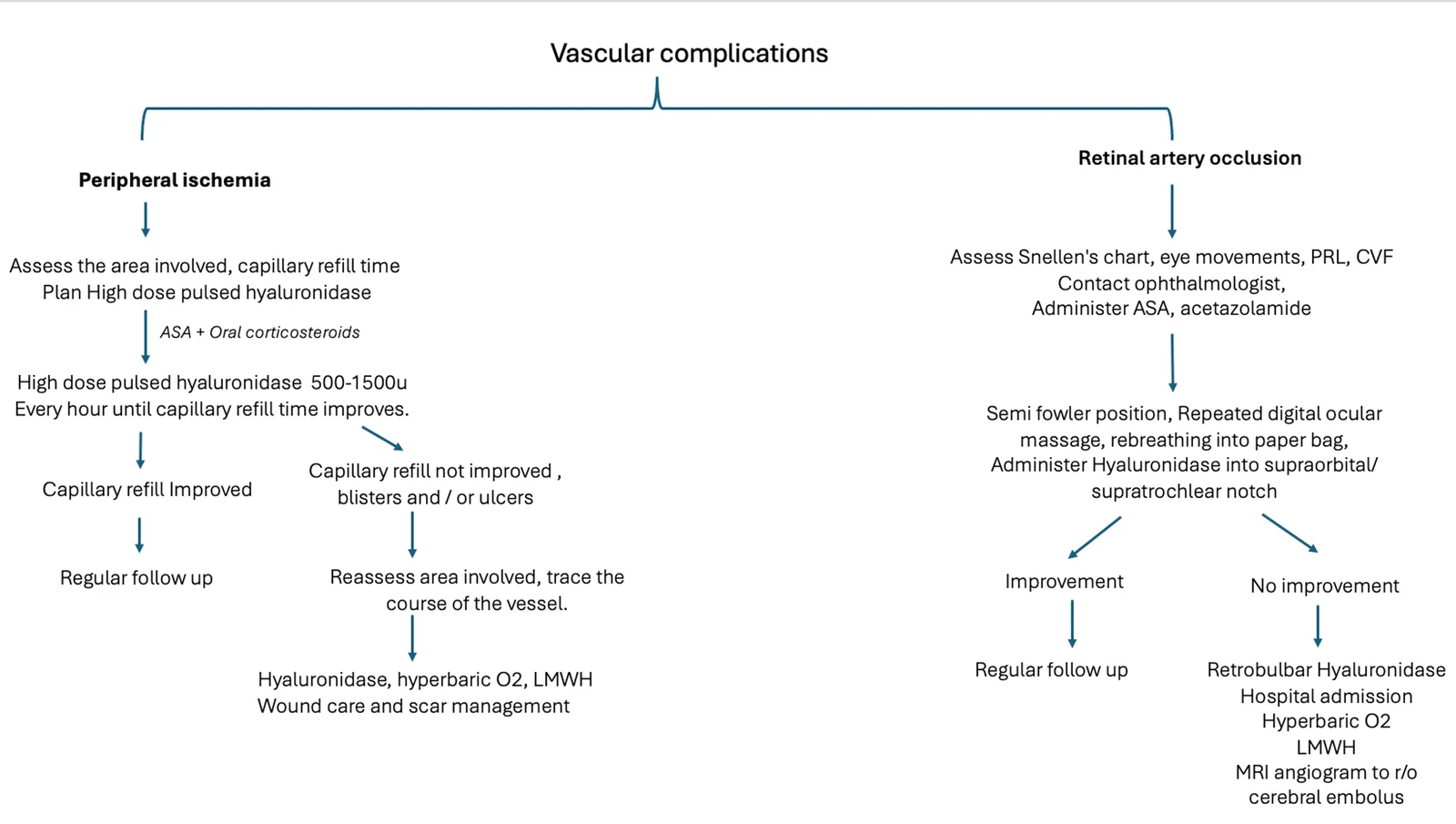
Vascular complications of hyaluronic acid fillers and their management. LMWH: low-molecular-weight heparin 1.5 mg/kg/day; PRL: pupillary light reflex; CVF: confrontation visual fields; ASA: acetylsalicylic acid

**Figure 9:**
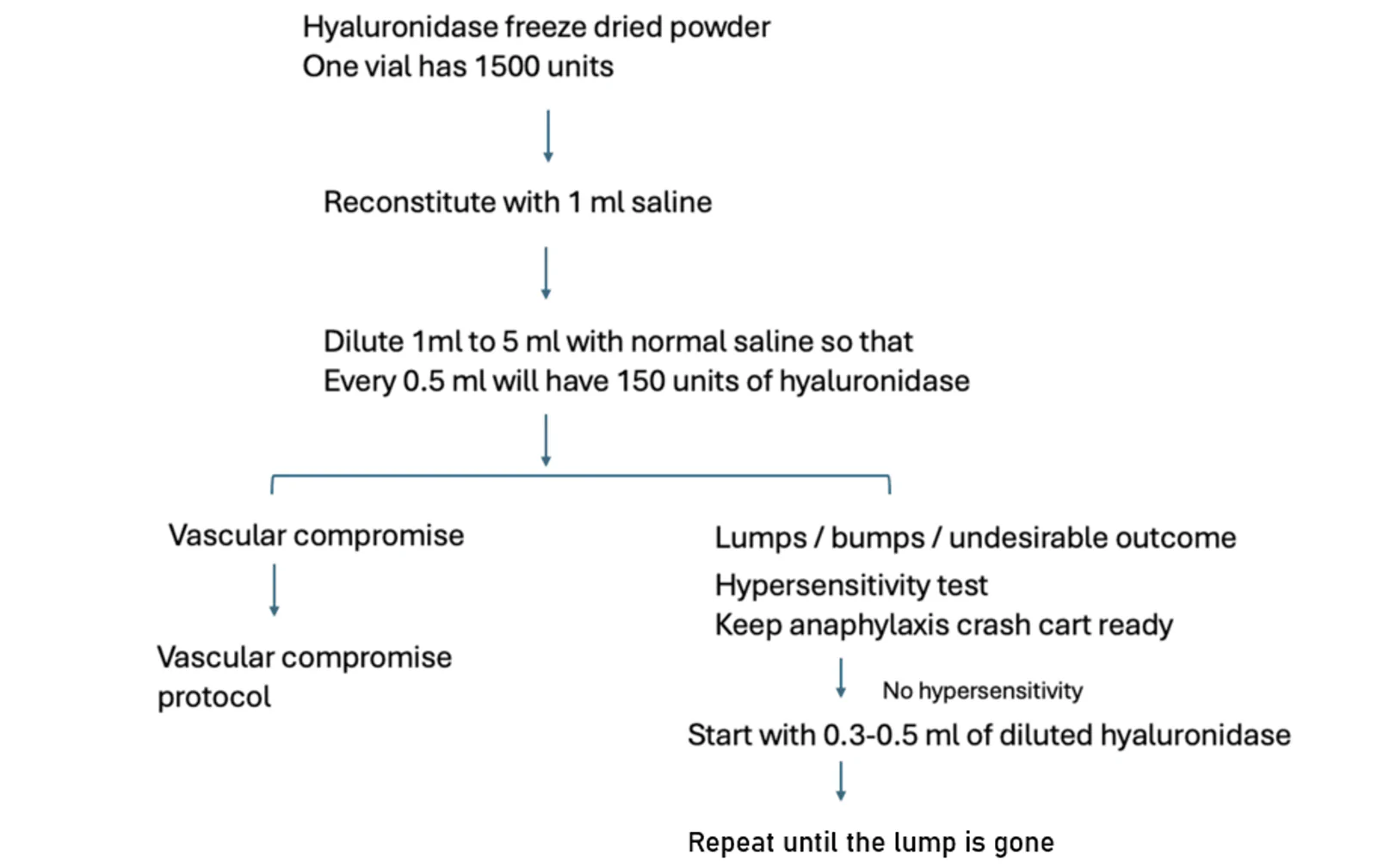
Protocol for administration and reconstitution of hyaluronidase.

On the following day, the patient should be reassessed for reactive hyperemia and capillary refill time, with repeat administration of HYAL if capillary refill remains prolonged or does not show improvement. Ocular assessment should include visual acuity using the Snellen chart, pupillary light reflex, extraocular movements, and evaluation for eyelid ptosis. Confrontation visual fields should also be assessed to identify any visual field deficit [48,53-56].

Overfill Syndrome

Facial overfill syndrome (FOS), sometimes referred to as “pillow face,” occurs when excessive or improperly placed HAFs produce disproportionate facial volume and distortion of natural facial contours [57,58]. Clinical manifestations include excessive midfacial volume, an unnatural smile, and a bloated or puffy appearance. It may result from excessive filler volume, repeated treatment without adequate reassessment, inappropriate injection technique, or aggressive treatment without sufficient consideration of underlying facial anatomy [57,58].

Prevention requires a conservative “less is more” approach, comprehensive assessment of facial proportions and tissue quality, appropriate product selection, and realistic counseling regarding treatment expectations. Patients with pre-existing skin laxity or structural changes should be counseled regarding alternative treatment options, including energy-based devices (EBDs), radiofrequency-based treatments, or high-intensity focused ultrasound where appropriate [57,58].

Management depends on the degree and location of overfilling. For HA-related overcorrection, multiple staged sessions of HYAL, preferably with ultrasound guidance where available, may be required rather than attempting complete correction in a single session. Physical modalities such as radiofrequency or high-intensity focused ultrasound may be considered selectively to address residual soft-tissue laxity after correction. Patient counseling and psychological support may also be appropriate, particularly in individuals with significant dissatisfaction or repeated procedures related to unrealistic treatment expectations [57,58].

Overall, complications associated with HAF injections range from transient and self-limiting reactions such as bruising and edema to delayed inflammatory nodules, infection, facial overfilling, and rare but potentially devastating vascular and ocular complications [48,54,57-59]. Prevention relies on careful patient selection, detailed knowledge of facial anatomy and vascular pathways, appropriate product selection, conservative dosing, accurate injection depth, meticulous aseptic technique, and early recognition of adverse events. Management should be tailored to the underlying mechanism and severity, with immediate specialist referral for serious complications such as vascular compromise and acute visual loss [48,54,57-59].

Factors influencing the risk of filler-related complications

The risk of filler-related complications is influenced by patient-, product-, and physician-related factors, all of which should be carefully evaluated before treatment [60,61]. Patient-related factors include a thorough medical history to identify active autoimmune disorders, such as systemic lupus erythematosus (SLE) and rheumatoid arthritis, active systemic or localized infections (including herpes simplex), recent dental procedures, and any previous surgical or non-surgical aesthetic treatments that may have altered the regional anatomy [60,61].

Furthermore, a detailed allergy history and a detailed medication history should identify anticoagulant and antiplatelet therapy, NSAIDs, and supplements (e.g., garlic, high-dose vitamin E, and *Ginkgo biloba*), with potential effects on hemostasis. Medically indicated antithrombotic therapy should not be routinely discontinued solely to reduce bruising associated with minimally invasive aesthetic procedures. Any modification should be based on individual thrombotic and procedural bleeding risk and, where appropriate, discussed with the prescribing physician [60-64].

Makeup use should also be considered, as it may contribute to the delayed development of nodules. Strict aseptic precautions, including thorough cleansing of the treatment area before injection, are essential. Following the procedure, patients should initially avoid makeup or use only new, previously unused products to minimize the risk of infection and DONs [60,61,65].

Clinical examination should assess for any disruption of the skin barrier, active cutaneous infections, or potential biofilm sources, such as acne or rosacea, at the planned injection sites. The presence of pre-existing filler material should also be documented. A comprehensive facial assessment using clinical examination, standardized photography, facial measurements, and validated assessment tools (e.g., the Facial Aesthetic Scale (FAS)) helps align patient expectations with realistic treatment outcomes. In addition, documenting baseline facial asymmetry and performing psychological screening to identify body dysmorphic disorder are important components of pre-procedural assessment. Finally, informed consent should be obtained using a signed consent form in the patient's preferred language, clearly explaining the procedure, expected outcomes, and potential complications [60,61].

Product-related factors primarily involve the rheological properties of the filler, including the degree of cross-linking, HA concentration, elastic modulus (G′), viscous modulus (G″), tan delta (tan δ), gel swelling characteristics, tissue integration, flexibility, and biocompatibility. These properties influence the filler's behavior within tissues and consequently affect both treatment outcomes and the likelihood of complications [60,61].

Physician-related factors include the injector's knowledge of facial anatomy, clinical experience, patient selection, and proficiency in injection techniques. Careful treatment planning, appropriate product selection, and meticulous injection technique are critical in minimizing the risk of adverse events and achieving optimal aesthetic outcomes [33,60,61].

Thread lifts

Complications of thread lifts include the following [66-69].

Infection

Thread lift, as any other surgical procedure, needs strict asepsis [66]. Since the entry port in cog threads is often close to or within the hairline, asepsis is more important. Systemic antibiotics in the form of amoxicillin-clavulanic acid 625 mg TID or clindamycin 300 mg TID may be considered for 7-10 days. Inadequate treatment leads to abscess formation and must be dealt with adequately [66-69].

Pain

Procedure pain can be minimized with adequate local and surface anesthesia. It is recommended that for local anesthesia, lignocaine 2% with adrenaline be used to ensure good access, free from bleeding. The correct placement depth of cog threads is the subcutaneous fat. Pain or excessive discomfort while advancing the cog threads is a sign of incorrect depth. Lignocaine injection along the tract helps reduce pain in thicker threads [66,67].

Bleeding

Certain regions such as the nose are more likely to have a vascular complication. Another case of bruising in the breast was seen after thread lift for breast ptosis (Figure 10) [70].

**Figure 10:**
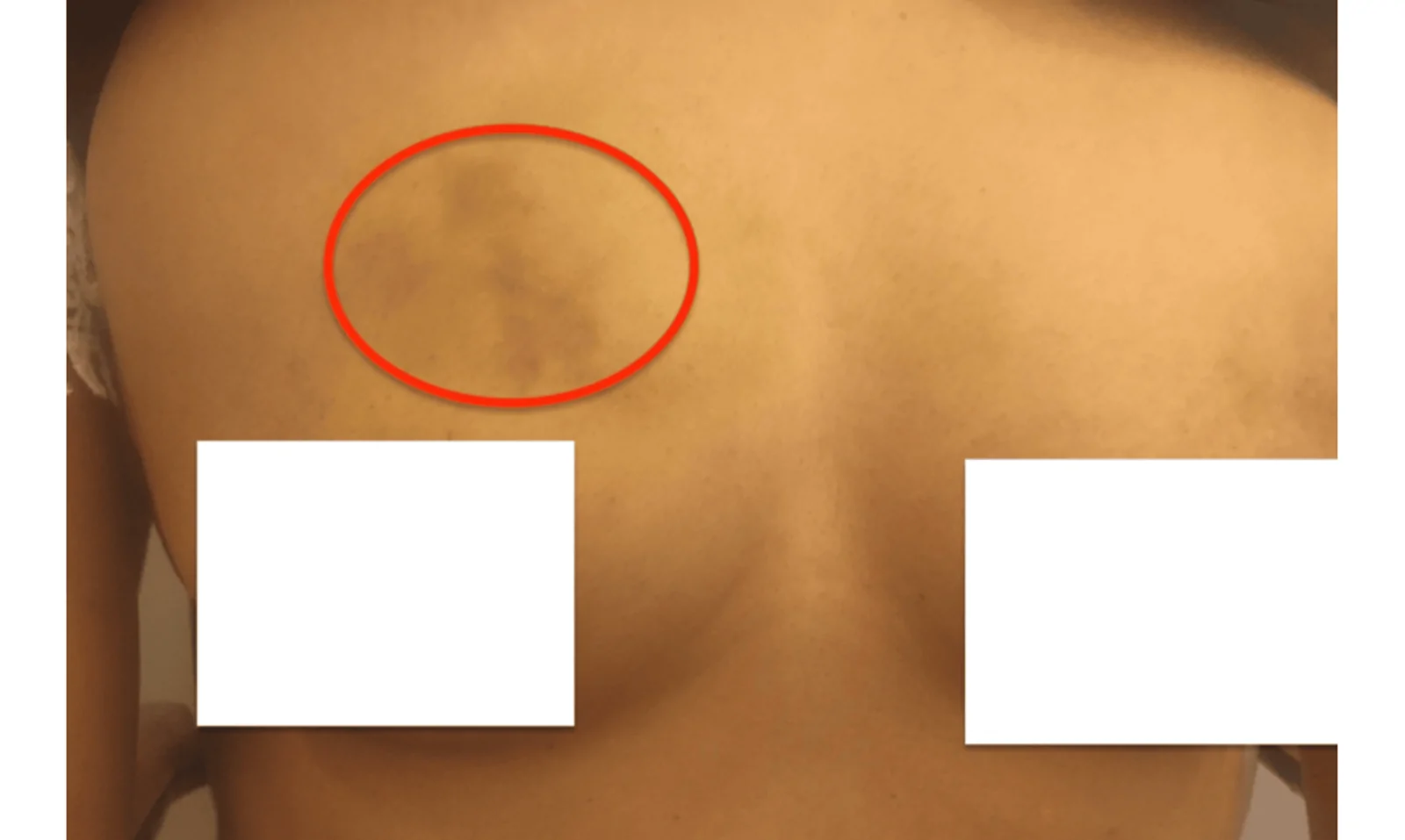
Bruising after thread insertion for breast ptosis (red circle). Written informed consent for publication was obtained from the patient.

In case of bleeding or hematoma at the entry point, manual pressure at the site and ice packs help prevent bruising. The site must be abandoned, and a fresh access be sought. Arnica™ or vitamin K-based creams help resolve the bruise faster [70]. Double-needle threads, being more traumatic, may lead to vessel injury. Passing the cog thread through a hematoma should be avoided, as this leads to a linear bruise along the thread tract [70].

Dimpling and Puckering

Superficial placement may lead to dimpling and puckering of the skin (Figure 11), which, if unattended, may lead to fibrosis [70]. Withdraw the thread and reintroduce it at the correct depth.

**Figure 11:**
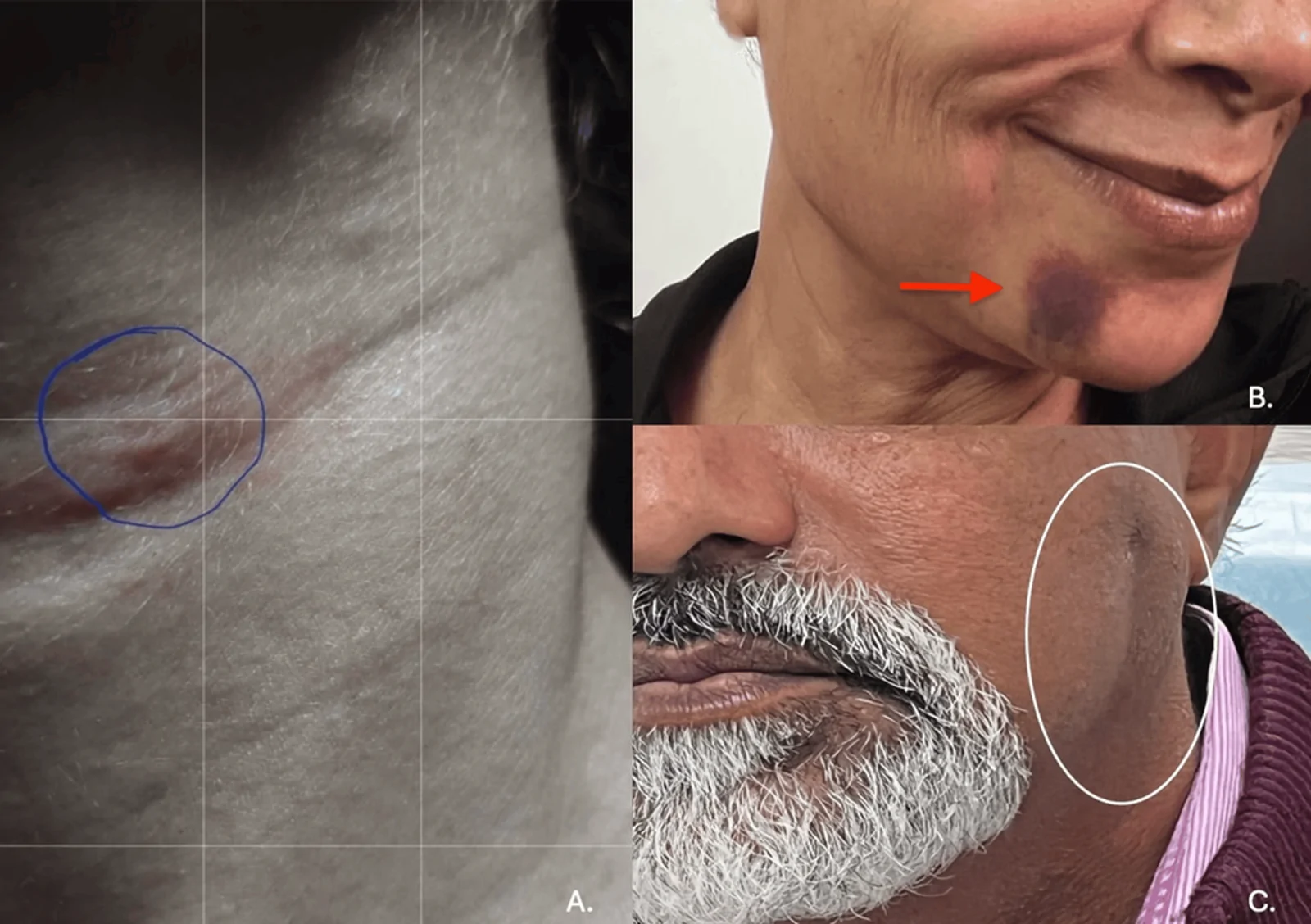
(A) Superficial entry and insertion of dermal threads for neck rejuvenation with a papule at the entry point and visible thread (blue circle). (B) Dimpling at the exit point due to superficial exit and bruising after the insertion of double-needle threads (red arrow). (C) Puckering after insertion of cog threads due to superficial placement (white circle). Written informed consent for publication was obtained from the patients.

Sometimes, dimpling is due to the tethering of the skin to the barbs, and this may ease out spontaneously within two to seven days. In such cases, “untethering” the thread may be attempted with a subcision around the area of puckering and releasing the thread attached to the tissue. If subcision is not successful, one may resort to injecting saline into the sunken area followed by massage or injecting a soft-tissue filler to smooth out the surface [66]. If dimpling persists with the above methods, surgical removal of the thread may be considered [66,70].

Thread Prolapses, Protrusion, and Migration

Monothreads may prolapse if not placed in their entire length at the correct depth. A prolapsed thread is best removed by withdrawing it at the protruded end. Cog threads, if not buried after snipping the ends, shall protrude from the entry point. This may lead to granuloma formation if left unattended [71,72]. It is best prevented by ensuring the knot or twisted ends are well buried while re-draping the skin. Thread migration is a complication of monothreads. Excessive movement or facial massage/rubbing can result in thread migration. In areas with flabby or loose skin, they may even bunch up, necessitating their removal. Ensuring secure fixation is crucial to prevent migration, particularly in dynamic areas such as the mouth and forehead, where botulinum toxin may be used to reduce muscle activity and stabilize thread positioning [71,72].

Prominence of Folds, Malar Eminence, and Asymmetry

Assess and mark vectors with the individual in an upright position. Reclining causes a shift in tissue, resulting in an asymmetrical outcome or inadequate procedure result. Extending the placement of cog threads beyond a fold results in skin bunching at the crease, resulting in the prominence of the folds and inadequate effacement [71,72].

The complications, mechanism, prevention, and management strategies associated with the use of facial threads have been tabulated and shown in Table 1 [66-72].

**Table 1:** Complications associated with threads [66-72]. ILS: intralesional corticosteroid; NSAIDs: non-steroidal anti-inflammatory drugs; FB: foreign body

Complication	Mechanism	Prevention	Management
Pain during insertion	Incorrect plane/inadequate anesthesia	Infiltration with lidocaine 2% + adrenaline; ensure correct plane	Reassure; reinfiltrate anesthesia if needed
Infection	Bacterial contamination	Strict asepsis; avoid going through inflamed skin	Antibiotics × 7 d; drain abscess if needed
Dimpling/puckering	Superficial placement; tethering	Correct vector and depth; adequate number of threads	Subcision/untethering; remove if persistent
Thread migration	Incorrect vectoring/loose fixation	Accurate markings; correct anchoring	Remove if symptomatic
Thread protrusion/extrusion	Shallow placement or poor quality threads	Ensure depth and anchoring; avoid over-retraction	Trim protruding end; remove if extruded or infected
Hematoma	Vascular injury during insertion	Gentle handling, blunt cannula threads	Cold compresses, oral NSAIDs, warm compresses after 24 hours
Nerve injury (transient paresthesia)	Deep placement close to nerve bundles	Anatomical precision, respect danger zones	Reassurance, usually self-limiting; refer to neurologist if persisting beyond 3-6 months
Granuloma/FB reaction	Host immune response/biofilm formation	Strict asepsis, certified threads	ILS, antibiotics, excision

Strengths and limitations

This review provides a comprehensive overview of complications associated with botulinum toxin, dermal fillers, and thread lifts, integrating available evidence with practical recommendations for their prevention, recognition, and management. A major strength is the emphasis on clinically relevant complications and their management across commonly performed aesthetic procedures. However, as this was a narrative rather than a systematic review, the literature search and selection process were not conducted according to a formal systematic review framework, and formal risk-of-bias assessment was not performed. The available evidence is also heterogeneous with respect to study design, patient populations, treatment techniques, doses, and definitions of adverse events. For rare or severe complications, high-level evidence is often limited; therefore, some recommendations necessarily rely on published clinical experience and expert consensus, and thus, selection and publication bias cannot be excluded.

Future directions

Future research should focus on prospective multicenter studies to establish more robust estimates of the incidence, risk factors, and outcomes of aesthetic complications. Development of standardized definitions and severity-grading systems, procedure-specific prevention protocols, and evidence-based algorithms for early recognition and management would facilitate more consistent clinical practice. Structured training and competency assessment for aesthetic practitioners, together with standardized documentation and longitudinal reporting of adverse events, may further improve procedural safety and patient outcomes. Greater evidence regarding long-term outcomes and comparative effectiveness of different management strategies is also warranted.

Medicolegal considerations

From a medicolegal perspective, appropriate patient selection, detailed documentation of baseline anatomical and functional findings, and comprehensive informed consent are essential components of safe aesthetic practice. Patients should be counseled regarding expected outcomes, potential complications, their severity and reversibility, and available management options. Accurate documentation of the product used, dose, treatment site, injection technique, and relevant patient-related factors is important. Prompt recognition and appropriate management of complications, timely referral to relevant specialists when indicated, and thorough documentation of adverse events and their subsequent management are equally important for maintaining patient safety and reducing potential medicolegal disputes.

## Conclusions

Minimally invasive aesthetic procedures, including botulinum toxin injections, HAFs, and thread lifts, have transformed facial rejuvenation by offering effective treatment with minimal downtime. However, despite their favorable safety profile, complications ranging from transient aesthetic concerns to vision-threatening vascular events may occur. A comprehensive understanding of facial anatomy, meticulous patient selection, appropriate product selection, and adherence to sound injection principles remain the cornerstones of preventing adverse events. Early recognition of complications and prompt, evidence-based management are critical to minimizing morbidity and optimizing patient outcomes. This review provides a practical overview of the mechanisms, prevention strategies, and management algorithms for the most commonly encountered complications associated with these procedures. Adoption of standardized treatment protocols, regular anatomical training, and preparedness to manage emergencies will further enhance patient safety and improve clinical outcomes in aesthetic practice.
